# On the Barometer

**Published:** 1811-02

**Authors:** Richard Walker


### *On the Barometer. By* Richard Walker, *Esq.*

(Continued from p. 52 of our last.)

(Phil. Mag.)

A *warm temperature* of the air, at any degree of density of the atmosphere, will retain a greater portion of water in a state of *chemical combination*, than a *cold temperature* of the air at a similar degree of density of the atmosphere. Hence we may account for the almost constant *dry state* of the lower stratum of the atmosphere during the SUMMER SEASON, and the almost constant *moist state* of the lower stratum of the atmosphere during the WINTER SEASON; the air, however, being sometimes sufficiently dense, as in the clear weather which accompanies a freezing atmosphere, to retain the water in a state of *chemical combination*, notwithstanding the diminution of temperature.

The same circumstance accounts likewise for the different states, with respect to *moisture and dryness*, of the middle seasons, viz. SPRING AND AUTUMN, accordingly as these participate in their nature more or less of either of the former seasons; observing that, *cæteris paribus*, there is more rain and misty weather during AUTUMN than SPRING, in consequence of the greater quantity of water which has been raised into the atmosphere during the SUMMER than the WINTER SEASON.

All the circumstances I have had occasion to mention, depending upon the *greater or less density*, and the *higher and lower degrees of temperature* of the atmosphere, are exemplified by the two following familiar experiments:

In the first instance, by means of pumping out of a glass receiver (containing air apparently dry and perfectly transparent) a certain portion of the air it contains, when the air being rarefied deposits a certain portion of the water it originally contained in *chemical combination* in a cloudy vapour, which, upon re-admission of the air, is re-absorbed; and in the second instance, by abstracting heat from a glass vessel containing atmospherical air, and again restoring the heat. The latter circumstance is likewise instanced, *naturally*, by what commonly happens in the course of a hot summer's day, particularly when the ground has become very moist by previous rain; the vapour *ascending* visibly in the morning, disdisappearing during the middle of the day, and *descending* visibly again in the evening.*

The variations of temperature in the atmosphere, *independent of those which proceed from the direct influence of the sun*, arise from the conversion of water into vapour, which produces *cold;* and the condensation of vapour into water, which produces *heat*. Hence it commonly follows, that in proportion as the barometer *rises*, the thermometer *sinks*, and *vice versa*, throughout the year; the direct influence of the sun in *clear weather* being abstracted†.

Thunder frequently follows a considerable duration of dry hot weather, both these circumstances being favourable to the collection and insulation of electric matter.

---

The extraordinary *elevation* of the barometer which sometimes happens, is said to arise from two currents of air, from opposite directions, meeting and accumulating over a particular spot; and the extraordinary *depression* of the barometer, from the circumstance of two currents of air setting out from any particular spot: in either case a commotion of the air is necessarily produced, whilst the equilibrium is restoring.

That the atmosphere, as well as the sea, is affected periodically in a small degree by the attraction of the moon, is well ascertained; but it does not appear that the weather is in the least influenced by any mechanical effect of the moon.

I was led to remark a difference of the weather during the *increase* and during the *wane* of the moon, by observing that eclipses of the moon were much seldomer obscured by a cloudy atmosphere than eclipses of the sun; and subsequent observations of a general nature have somewhat confirmed me in the same opinion.

---

### *For the Medical and Physical Journal.*

### *Observations on White Serum.*

WE have been favoured, by JOSHUA BROOKS, ESQ. (Theatre of Anatomy, Blenheim Street) with a portion of SERUM *of Human Blood*, which in appearance exactly resembles milk.

---

* At Lima, in Peru, it never rains; the moisture in the day time being restored again at night in the state of mist.

† In summer, during fair weather, the nights, or rather the mornings before sun-rise, are cold, approaching even to frost.

